# Editorial: Impact of landscape and feeding on the bees’ gut microbiome shaping and pathogens development

**DOI:** 10.3389/fcimb.2025.1688273

**Published:** 2025-10-10

**Authors:** Daniele Alberoni, Diana DiGioia

**Affiliations:** Dipartimento di Scienze e Tecnologie Agro-Alimentari, University of Bologna, Bologna, Italy

**Keywords:** microbiome, pathogen dynamics, landscape simplification, feeding practices, floral resources, climate change

Bees are pivotal for the stability of ecosystems and agricultural productivity, yet their health is constantly challenged by a variety of stressors. Among these, impactful bee pathogens such as *Nosema* spp., *Crithidia* spp., and numerous viruses have spread globally, facilitated by the international trade of pollinators (Smith et al., 2013; Graystock et al., 2013), but also by spillover and spillback dynamics between wild and managed pollinators in natural and anthropogenic landscapes (Proesmans et al., 2021). Pathogen spread is further promoted by landscape simplification and intensive agricultural practices that reduce the availability and diversity of floral resources (Figueroa et al., 2020) and biodiversity. In such agroecosystems, the limited floral resources available may be contaminated with pathogens (Figueroa et al., 2019), contributing to a faster pathogens spread. In this context, the spread of bee pathogens may occur directly through contact with contaminated habitats, or indirectly via biodiversity loss that alters the host–pathogen interactions (Fearon et al., 2023), thereby adding complexity to pollinator health dynamics.

Recently, the gut microbiome has emerged as a central determinant of resilience, providing essential functions for nutrition, immunity, and protection against pathogens (Raymann and Moran, 2018). Environmental changes and their impact on the forage resources, interact with host-associated microbial communities, influencing the bee fitness and their immune response (Braglia et al., 2025) but also the development and virulence of pathogens (Di Pasquale et al., 2013; Giacomini et al., 2018). Understanding these interactions is therefore crucial to address pollinator decline and safeguard ecosystem services.

Climate change further complicates this scenario. Altered flowering phenology and changes in the nutritional quality of pollen and nectar may compromise pollinator diets and the acquisition of beneficial microorganisms from the environment. These shifts can weaken host defenses and reduce their longevity, with cascading effects on crop production, wild plant reproduction, and ultimately global food security.

The articles collected in this Research Topic provide new insights into how nutrition, microbial balance, and environmental factors modulating bee pathogens development and the reciprocal influence between disease and microbiota. Collectively, they show that the gut microbiome does not act in isolation but is embedded within broader ecological networks that connect bees, their environment, and the pathogens they host.

Braglia et al. demonstrated that the opportunistic bacterium *Serratia marcescens*, although typically present at low levels in the bee gut, can cross the intestinal barrier and cause septicemia, with virulence exacerbated when co-infecting with *Vairimorpha (Nosema) ceranae* in laboratory conditions. The study further highlighted how environmental microorganisms such as *Apilactobacillus kunkeei*, often populating flowers and bee environments (Neveling et al., 2012; Garrido et al., 2024), may reduce both *Serratia* and *V. ceranae*, underscoring the role of microbial interactions in controlling pathogen development.

Wei and Huang focused on the evolutionary ecology of *N. ceranae*, comparing its infection in *Apis cerana* and *Apis mellifera*. Their inoculation experiments revealed a distinct trade-off: in *A. mellifera* the parasite proliferates extensively, producing high spore loads but causing relatively low host mortality, whereas in *A. cerana* it induces markedly higher mortality despite generating fewer spores. Transcriptomic analyses further showed a stronger suppression of host gene expression in *A. cerana*, particularly in immune pathways. These findings suggest that *N. ceranae* is adapted for high proliferation with low virulence in *A. mellifera*, while maintaining higher virulence but limited transmission potential in *A. cerana*, highlighting the complex trade-offs of parasite evolution in a multi-host system

Sgroi et al. provided an epidemiological overview from Italy, documenting the widespread occurrence of both *Nosema apis* and *N. ceranae* in bees, pollen, and honey. The study showed higher infection risks in high-impact areas subject to intensive anthropogenic pressures. Molecular analyses revealed that *N. apis* sequences were uniform, while *N. ceranae* exhibited four distinct sequence types characterized by single nucleotide polymorphisms, indicating high haplotype diversity but low nucleotide diversity. This genetic pattern points to recent population expansion and suggests ongoing adaptation of *N. ceranae* to local environments. The study underscores the strong link between pathogen prevalence and environmental quality, emphasizing the need for continuous surveillance and monitoring.

Finally, Weinhold et al. investigated the bumble bee *Bombus terrestris* and showed how outdoor exposure leads to a temporal succession in the gut microbiota, with increasing abundance of lactic acid bacteria and a parallel decline of core taxa such as *Snodgrassella* and *Gilliamella*. Interestingly, flower diversity per se did not strongly affect microbial progression, but environmental exposure was sufficient to trigger diversification. This work reinforces the concept that the gut microbiota is dynamically shaped by the environment and not merely by diet composition.

Collectively, the contributions in this Research Topic underscore that bee health cannot be understood in isolation from its ecological and nutritional context. Nutrition, feeding practices, and pathogen dynamics are tightly interlinked and modulated by environmental conditions. Rather than considering the landscape merely as a mosaic of floral resources, it becomes necessary to view it as an integrated system where climate, habitat management, feeding regimes, and microbial interactions converge to shape bee resilience.

Against this background, the microbiome of the gut becomes an intermediary, balancing landscape and diet impacts on immunity and susceptibility to pathogens. The experiments compiled here attempt to demonstrate that feeding and landscape simplification, or alterations in floral resources all have discernible impacts on microbial equilibrium and pathogen development, virulence and disease dynamics. They also point up the promise of microbial friends and feeding interventions in blunting pathogen pressure.

Taken together, this Research Topic contributes to demonstrate that bee resilience is inseparable from environmental integrity and nutritional quality. Feeding regime, microbial richness and pathogen evolution work together, impacting pollinator and ecosystem stability. Future studies should therefore shed light on microbiota–pathogen interaction under different feeding and landscape scenarios, and experimentally test interventions, from microbial augmentation to landscape management, able to ensure bee well-being under rapidly changing circumstances.
